# A Novel Automatic Algorithm to Support Lung Ultrasound Non-Expert Physicians in Interstitial Pneumonia Evaluation: A Single-Center Study

**DOI:** 10.3390/diagnostics14020155

**Published:** 2024-01-10

**Authors:** Marialuisa Sveva Marozzi, Sebastiano Cicco, Francesca Mancini, Francesco Corvasce, Fiorella Anna Lombardi, Vanessa Desantis, Luciana Loponte, Tiziana Giliberti, Claudia Maria Morelli, Stefania Longo, Gianfranco Lauletta, Antonio G. Solimando, Roberto Ria, Angelo Vacca

**Affiliations:** 1 Unit of Internal Medicine “G. Baccelli”, Department of Precision and Regenerative Medicine and Ionian Area (DiMePRe-J), University of Bari Aldo Moro Medical School, 70124 Bari, Italy; 2 Institute of Clinical Physiology, National Research Council, 73100 Lecce, Italy; 3 Pharmacology Section, Department of Precision and Regenerative Medicine and Ionian Area (DiMePRe-J), University of Bari Aldo Moro Medical School, 70124 Bari, Italy; 4 Interdepartmental Centre for Research in Telemedicine (CITEL), Department of Precision and Regenerative Medicine and Ionian Area (DiMePRe-J), University of Bari Aldo Moro Medical School, 70124 Bari, Italy

**Keywords:** automatic algorithm, interstitial lung disease, lung ultrasound

## Abstract

Introduction: Lung ultrasound (LUS) is widely used in clinical practice for identifying interstitial lung diseases (ILDs) and assessing their progression. Although high-resolution computed tomography (HRCT) remains the gold standard for evaluating the severity of ILDs, LUS can be performed as a screening method or as a follow-up tool post-HRCT. Minimum training is needed to better identify typical lesions, and the integration of innovative artificial intelligence (AI) automatic algorithms may enhance diagnostic efficiency. Aim: This study aims to assess the effectiveness of a novel AI algorithm in automatic ILD recognition and scoring in comparison to an expert LUS sonographer. The “SensUS Lung” device, equipped with an automatic algorithm, was employed for the automatic recognition of the typical ILD patterns and to calculate an index grading of the interstitial involvement. Methods: We selected 33 Caucasian patients in follow-up for ILDs exhibiting typical HRCT patterns (honeycombing, ground glass, fibrosis). An expert physician evaluated all patients with LUS on twelve segments (six per side). Next, blinded to the previous evaluation, an untrained operator, a non-expert in LUS, performed the exam with the SensUS device equipped with the automatic algorithm (“SensUS Lung”) using the same protocol. Pulmonary functional tests (PFT) and DLCO were conducted for all patients, categorizing them as having reduced or preserved DLCO. The SensUS device indicated different grades of interstitial involvement named Lung Staging that were scored from 0 (absent) to 4 (peak), which was compared to the Lung Ultrasound Score (LUS score) by dividing it by the number of segments evaluated. Statistical analyses were done with Wilcoxon tests for paired values or Mann–Whitney for unpaired samples, and correlations were performed using Spearman analysis; *p* < 0.05 was considered significant. Results: Lung Staging was non-inferior to LUS score in identifying the risk of ILDs (median SensUS 1 [0–2] vs. LUS 0.67 [0.25–1.54]; *p* = 0.84). Furthermore, the grade of interstitial pulmonary involvement detected with the SensUS device is directly related to the LUS score (r = 0.607, *p* = 0.002). Lung Staging values were inversely correlated with forced expiratory volume at first second (FEV1%, r = −0.40, *p* = 0.027), forced vital capacity (FVC%, r = −0.39, *p* = 0.03) and forced expiratory flow (FEF) at 25th percentile (FEF25%, r = −0.39, *p* = 0.02) while results directly correlated with FEF25–75% (r = 0.45, *p* = 0.04) and FEF75% (r = 0.43, *p* = 0.01). Finally, in patients with reduced DLCO, the Lung Staging was significantly higher, overlapping the LUS (reduced median 1 [1–2] vs. preserved 0 [0–1], *p* = 0.001), and overlapping the LUS (reduced median 18 [4–20] vs. preserved 5.5 [2–9], *p* = 0.035). Conclusions: Our data suggest that the considered AI automatic algorithm may assist non-expert physicians in LUS, resulting in non-inferior-to-expert LUS despite a tendency to overestimate ILD lesions. Therefore, the AI algorithm has the potential to support physicians, particularly non-expert LUS sonographers, in daily clinical practice to monitor patients with ILDs. The adopted device is user-friendly, offering a fully automatic real-time analysis. However, it needs proper training in basic skills.

## 1. Introduction

Lung Ultrasound (LUS) is broadly used in clinical practice [1]. Since the COVID-19 pandemic, LUS has received much attention [2]. Some studies have tried to relate the LUS score, which measures the severity of superficial lung disease [3], to other significant COVID-19 indicators for early decision-making. LUS has the advantage of an easy and quick assessment to identify and classify disease severity [4]. However, due to technical limitations, only subpleural spaces may be evaluated with LUS. Even if none of the LUS features is pathognomonic for a specific disease, much evidence supports its clinical value to evaluate treatment response both in intensive care and internal medicine departments [5,6,7], detect signs of pulmonary involvement, and disease progression or regression. 

Interstitial lung diseases (ILDs) are characterized by progressive lung fibrosis, mostly due to multiple interacting pathways [8]. ILDs encompass various types, each stemming from different underlying causes. A significant portion of ILDs have parallel autoimmune disorders (e.g., rheumatoid arthritis, systemic sclerosis, systemic lupus erythematosus, Sjögren syndrome) [9]. ILDs can be classified into different patterns considering the pulmonary images. The predominant patterns are Usual Interstitial Pneumonia (UIP) and Non-Specific Interstitial Pneumonia (NSIP) [10]. Despite High-Resolution Computer Tomography (HRCT) representing the gold standard imaging technique, LUS has high sensitivity and specificity for the diagnosis even in the early pre-clinical phase [11]. Accordingly, LUS applies in clinical practice to identify a given ILD and its evolution. In a daily clinical setting, LUS can be performed as a screening method or in follow-up after HRCT to evaluate typical patterns of interstitial lung involvement and monitor parenchyma outcomes. Proper training is needed to better identify typical lesions [12]. The most important findings in patients with ILDs during LUS are the number of B-lines, the alterations of the pleural line, and subpleural abnormalities [1].

Therefore, innovative AI and other automatic algorithms may help in this issue [13], as they can be trained/developed to identify and highlight abnormalities, lesions, or structures of interest in medical images, assisting operators in their interpretation [14].

In this context, we wondered whether the algorithm integrated into the “SensUS Lung” device, designed for the automatic recognition of the interstitial damage patterns, was effective in ILD recognition and scoring by an untrained physician—who was a beginner in ultrasonography, in particular in evaluating ILDs—compared to an expert LUS sonographer. The algorithm calculates an index that grades a possible interstitial involvement. As a secondary outcome, we investigated whether the SensUS evaluation was correlated with clinical respiratory evaluation.

## 2. Patients and Methods

### 2.1. Patients

The study included 33 Caucasian patients (17 M and 16 F, aged 69 ± 17 year) in outpatient follow-up for ILDs from 2021 to 2023 in the Unit of Internal Medicine “Guido Baccelli” of the Hospital Policlinico of Bari. Patients were evaluated during a medical visit in clinical practice. They were included in the protocol and signed informed consent. The study was conducted according to the Good Clinical Practice Guidelines of the Italian Ministry of Health and the ethical guidelines of the Declaration of Helsinki (as revised and amended in 2004), following the approval by the Ethics Committee of the University of Bari Medical School (Code ID no. 7010/2021 dated 13 October 2021).

For all patients, pulmonary functional tests (PFT) and diffuse lung carbon monoxide (DLCO) evaluation were available. Patients were excluded if chronic heart failure or current tumors were present.

### 2.2. Imaging

All patients presented typical HRCT patterns of ILDs (honeycombing, ground glass, fibrosis). Patients were included if HRCT was performed within three months before the evaluation planned for this study.

An expert physician evaluated all patients with LUS scores on twelve segments (six per side) as consolidated clinical practice in our institution [5]. Next, blinded to the previous evaluation, an untrained physician novice in LUS performed the exam using the “SensUS Lung” device and the same protocol. Both physicians were blinded to the patients’ HRCT reports.

### 2.3. Lung Ultrasound

**Figure 1:**
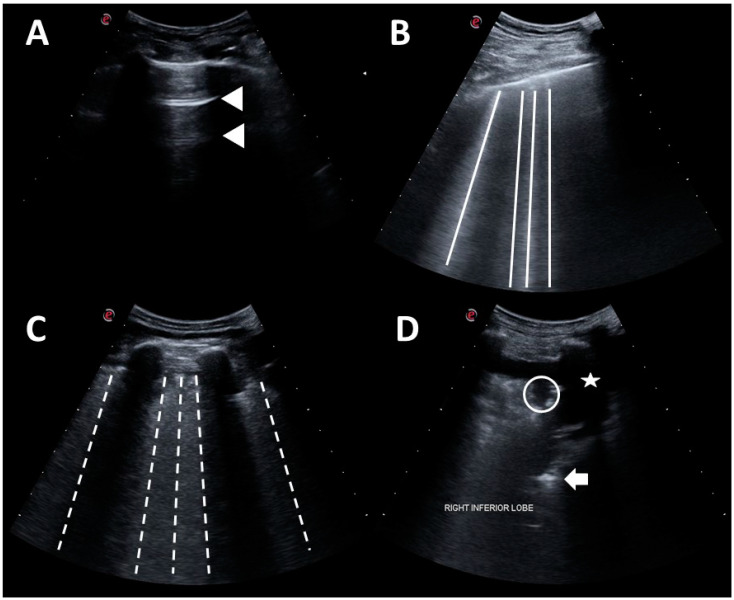
Lung Ultrasound Score was evaluated according to guidelines. (**A**) Score 0: normal pattern, A-lines or <3 B-lines; (**B**) Score 1: moderate loss, ≥3 B-lines; (**C**) Score 2: severe loss, coalescent B-lines; (**D**) Score 3: complete loss, white lung and/or lung consolidations. Legend: A-lines are indicated by triangles; B-lines are indicated by continue lines; coalescent B-lines are indicated by dashed lines; consolidation is indicated by circles; aerial bronchogram is indicated by an arrow; pleural effusion is indicated by stars (as reported in ref. [4]).

LUS was performed after the patients had rested for 10 min in a sitting position. A 5–12 MHz convex probe was used (Logiq E9 XClear 2.0, GE Healthcare, Chalfont St Giles, UK). The second harmonic wave was excluded, the depth was 10 cm, and the focus was placed on the pleural line. The LUS score was performed in a separate room, and the operator was blinded to any localization of the disease. The exam was performed using a 12-zone segmentation as already included in clinical practice [5]. The same operator performed all the LUS scores. According to the international guideline [12], the LUS score was evaluated on six segments for each lung, and each segment was scored as 0 for equal or less than 3 B-lines, 1 for more than 3 B-lines, 2 for B-lines more than 50% of the image without clear subpleural alterations, and 3 for white lung or consolidation (Figure 1).

### 2.4. Automated Algorithm

**Figure 2:**
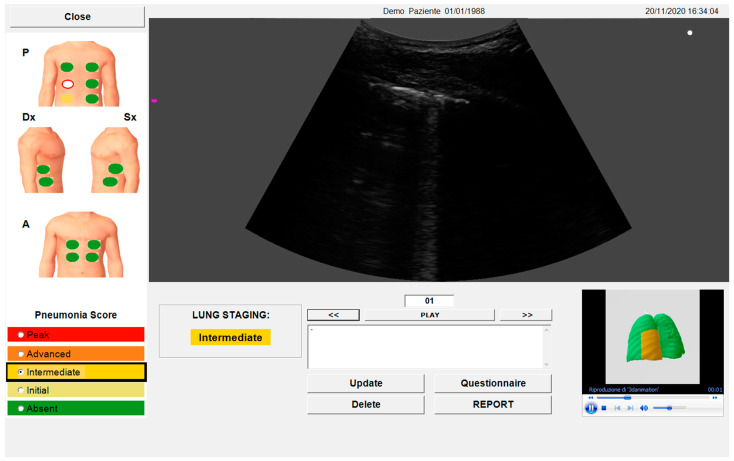
SensUS software interface after the acquisition. The image is a screenshot of the automatic data elaboration of the results, including the Pneumonia Score and Lung Staging.

An untrained and beginner ultrasonographer used the device equipped with an automated algorithm to evaluate the enrolled patients. The adopted device (SensUS Lung version 1.0, Amolab srl, Lecce, Italy) consisted of a battery-powered ultra-portable ultrasonographic unit and a convex probe operating at the nominal frequency of 3.5 MHz. The software module integrated into the device included a novel proprietary algorithm for the automatic recognition of the characteristic patterns of pneumonia, as described [15]. The lung acquisition was driven by the software (Figure 2), which had most of the pre-sets pre-configured with locked settings (e.g., tissue harmonics off). The operator may regulate depth, focus, and gain to obtain a better visualization of the pleura and lung.

A water-soluble and hypoallergenic coupling gel (Aquasonic^®^ 100 Ultrasound Gel, Parker Laboratories, Fairfield, NJ, USA) was used for the probe-skin coupling and sanitized before and after each use with disinfectant wipes. The acquisition protocol was fully guided by a software interface. To obtain a comparable evaluation, the lung acquisition with SensUS was applied using a 12-zone method as already validated in LUS [3], specifically, 6 zones per side (left and right). Each acquired zone was scanned with the probe in longitudinal and transversal direction, with 12 frames acquired per direction (thus, a total of 24 frames per zone were acquired).

The working principle of the adopted device is described below [15]. The algorithm analyses all frames through specific morphological filters and thresholds based on the geometrical distributions of the pixels in all images to recognize the ILD patterns automatically.

Then, the presence of the following signs was automatically detected by the algorithm: focal, multifocal, confluent B-lines or “lung comets”; small or consistent consolidations (in particular: small multifocal, intralobular or interlobular with possible dynamic aerial bronchogram consolidations); A-lines; pleural effusions. The algorithm assigns a score, named Pneumonia Score, associated with the examined anatomical zone, ranging from 0 to 4, based on the identified pneumonia signs. Finally, a total score for the patient, named Lung Staging, was assigned, ranging from 0 to 4 based on the maximum of the Pneumonia Score.

The “SensUS Lung” device indicated well-defined grades of interstitial involvement (absent, initial, intermediate, advanced, and peak). Lung Staging scored from 0 (absent) to 4 (peak). Therefore, to compare the Lung Staging measured with “SensUS Lung” to the LUS score (scored from 0 to 3) that was derived by an expert physician, the LUS total score (named crude LUS score) was divided by the number of segments evaluated (named mean LUS score). To have a comparable Lung Staging (measured with SensUS) with the LUS score, we reported the device scores from 0 (absent) to 3 (which includes advanced and peak). 

### 2.5. Flow-Volume Spirometry and Diffusion Lung Carbon Monoxide

A pulmonary functional test was performed with flow-volume spirometry at the time of the LUS evaluation during a medical visit, according to a standardized protocol [16]. Distal airflow obstruction was evaluated as forced expiratory volume at first second (FEV1%), forced vital capacity (FVC%), and forced expiratory flow (FEF) at the 25–75 percentile (FEF25–75%) of total flow. The same operator performed all flow-volume spirometry and validated the flow-volume spirometry evaluation. Flow-volume spirometry was performed with MicroLab portable (CareFusion, San Diego, CA, USA), and all results were processed by Spirometry PC Software version 3.20 (Vyaiere Medical). A diffusion lung carbon monoxide (DLCO) test was performed in another center to evaluate the grade of interstitial disease. Results were given within a month of the evaluation. They were categorized into reduced or preserved DLCO, according to the value of the test: “reduced” if DLCO was less than 60%, while “preserved” if it was equal or over 60%.

### 2.6. Statistics

Data were analyzed using SPSS version 21.0 (IBM, Armonk, NY, USA) and expressed as means ± S.D. for parametric data and median and interquartile range [IQR] for non-parametric ones. The distribution of dichotomous values was analyzed with a Chi-square test. As far as non-normally distributed data is concerned, we performed a non-parametric Mann–Whitney test for comparisons and Spearman distribution for correlations. Normally distributed data were studied with parametric unpaired *t*-test for comparisons and Pearson distribution. Statistical significance was set to *p* < 0.05.

## 3. Results

### 3.1. Patients

Patients were slightly overweight, with the majority being non-smokers, only one being a current smoker, and 27% having a history of smoking. About 42% presented a post-COVID ILD, while the remaining were affected by ILD without a history of SARS-CoV-2 infection (Table 1). Among the latter group, 21% (6 patients) were Non-Specific Interstitial Pneumonia (NSIP) attributed to autoimmune diseases (1 vasculitis, 1 systemic lupus erythematosus, 1 Sjögren syndrome, 1 systemic sclerosis) or systemic granulomatosis (3 sarcoidosis). Three patients had Organized Pneumonia, and the remaining 10 patients were affected by a Usual Interstitial Pneumonia (UIP) pattern due to secondary causes (1 to amiodarone, 3 to radiation therapy, 6 to professional exposure). None of the participants were in treatment with ILD-specific drugs, as their lung disease was considered stable both as clinical and radiological evaluation by a third referral pneumological center.

No differences were found in PFT values between all patients when they were classified according to the DLCO result, apart from a significantly lower forced vital capacity (FVC%) in patients with reduced DLCO (Table 2), as expected.

### 3.2. Algorithm Evaluation Compared to Human Evaluation

**Figure 3:**
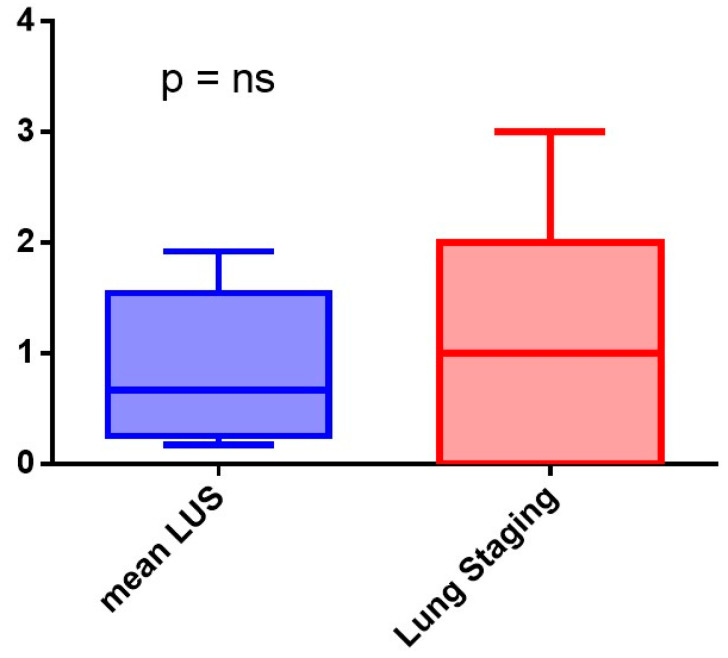
Comparison between mean Lung Ultrasound Score (LUS score) and Lung Staging measured with SensUS.

Lung Staging measured by SensUS demonstrated its capability to effectively identify the risk of ILD with a result statistically no different from that of the LUS score. The median Lung Staging was 1, with a range from 0 to 2, while the median LUS score was 0.67, ranging from 0.25 to 1.54; the difference was not statistically significant (*p* = 0.84). These findings suggest that Lung Staging can be a reliable alternative to the LUS score for the ILD risk assessment (Figure 3).

**Figure 4:**
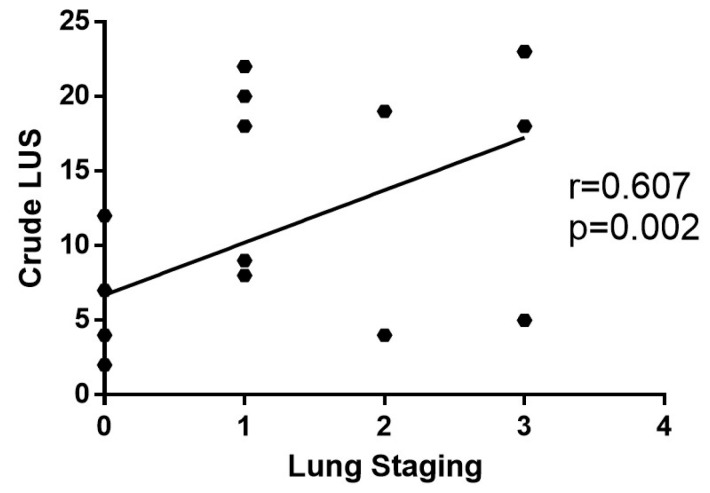
The direct correlation between *Lung Staging* measured with SensUS and crude Lung Ultrasound Score (LUS score) was statistically significant.

The degree of interstitial pulmonary involvement, as measured by the expert ultrasonographer with LUS score, exhibited a direct correlation with the extent of lung abnormalities evaluated with the Lung Staging (r = 0.607; *p* = 0.002) (Figure 4).

### 3.3. Clinical Meaning of the SensUS Lung Device Evaluation

**Figure 5:**
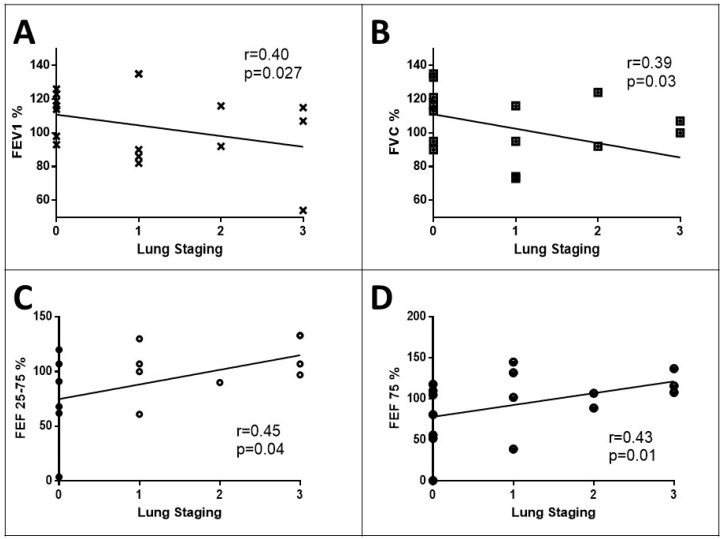
Correlation between Lung Staging measured with SensUS and pulmonary functional test (PFT) parameters in all patients as BMI, age, gender, and smoking habits. Lung Staging inversely relates to forced expiratory volume at the first and second (FEV1—panel **A**) and forced vital capacity (FVC—panel **B**); on the contrary, Lung Staging directly relates to forced expiration flow between the 25th and 75th percentile of evaluation (FEF 25–75%—panel **C**) and forced expiration flow at 75th percentile of evaluation (FEF 75%—panel **D**).

The study revealed important relationships between imaging results and lung function parameters. Considering patients as a whole, the Lung Staging was inversely correlated with forced expiratory volume at the first second (FEV1% r = −0.40, *p* = 0.027) and forced vital capacity (FVC% r = −0.39, *p* = 0.03). This indicates that Lung Staging may not only be effective in assessing the risk of ILDs but could also provide valuable clinical insights into the evaluation. In fact, considering Lung Staging as a measure of ILD severity, as it increases, lung function tends to decline. However, Lung Staging exhibited direct correlations with FEF25–75% (r = 0.45, *p* = 0.04) and FEF75% (r = 0.43, *p* = 0.01), indicating that these parameters may reflect different aspects of the lung function in the context of ILDs (Figure 5).

**Figure 6:**
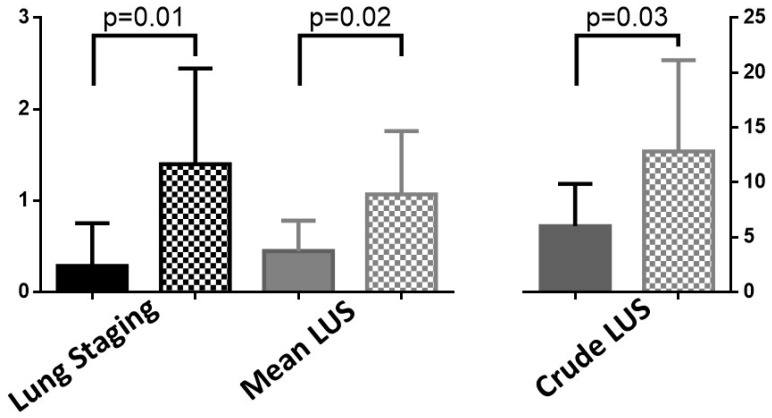
Lung Staging and Lung ultrasound score (LUS), both as mean and crude, in patients with preserved (plain) or reduced (dotted) DLCO.

Lastly, the study pointed out that in patients with reduced DLCO, Lung Staging classifications were significantly higher compared to those with preserved DLCO (reduced median 1 [1–2] vs. preserved 0 [0–1], *p* = 0.001). The overlap in results with the LUS score in this subgroup underscores the diagnostic efficacy of Lung Staging (reduced median 18 [4–20] vs. preserved 5.5 [2–9], *p* = 0.035). This is particularly true in cases where conventional lung function tests may be inconclusive, supporting a pneumologist non-expert in LUS to have the imaging evaluation of ILD severity (Figure 6).

## 4. Discussion

The fields of application of AI are varied, but the most significant challenge in the use of these algorithms in healthcare could be the difficulty of capturing and representing contextual information. For example, the process of anamnesis is difficult to fix in an automated manner; this issue becomes even more evident in patients with multiple comorbidities, where different aspects of the condition emerge progressively as more exams are performed, and results become available. Diagnostic evaluation is the main clinical application for AI, ranging from pathology to imaging [13,14,17,18,19,20]. Several authors have studied the impact of ultrasound on clinical management and patient outcomes in recent years, in particular since the COVID-19 pandemic [21,22,23].

Ultrasound has influenced healthcare in diagnostic and real-time imaging, guidance for procedures, improved surgery, emergency medicine, cardiovascular application, neurological assessment, and monitoring therapies. Moreover, portable and point-of-care ultrasound has expanded its use not only in emergencies but also in rural healthcare and developing countries [1]. The first claim about LUS is the reliability of the results. Numerous studies tested multiple methods to find the most affordable method to examine LUS [24]. Soldati et al. studied the sensibility and specificity of LUS in the diagnosis of pneumothorax. When compared to chest X-rays, LUS showed higher sensitivity and sensibility than computed (CT) tomography scans [25]. Of course, the reliability of this technique depends on the instrument used. A hand-held ultrasound has been proven to have a lower quality than other conventional ultrasound machines but can be used for specific indications.

Recently, AI has been applied in multiple fields. For example, new data about AI applied to ultrasound and echocardiography to enhance performance in time-sparing settings are being published. In emergency departments, the impact of this assessment would be greater if current measurements were automated [26]. A fully automated machine-learning algorithm could assess the extent of ventricular contraction without the initial requirement of identifying endocardial boundaries or measuring left ventricular volumes at end-systole and end-diastole [27,28]. The use in ILD patients was mostly as a diagnostic tool supporting CT scan imaging, as well as in quantitative parameters or the quantification of parenchymal lesions [29]. Recent reviews highlight the strengths and weaknesses of current developments in AI methods to detect ILD diagnosis and prognosis, underlining the need for several databases to improve and develop current data [30,31].

Walsh et al. tested an algorithm for systematic, objective fibrotic imaging analysis (SOFIA) vs. radiologist UIP probabilities. When prognostic accuracy was evaluated in the identification of the UIP pattern, only SOFIA predicted survival [17]. Furthermore, Furukawa et al. demonstrated the diagnostic accuracy of an AI algorithm that especially improved the accuracy of the diagnosis of idiopathic pulmonary fibrosis (IPF) when clinical data were also included in the evaluation [32]. In pathology, AI has been tested as well to predict UIP. Uemagi et al. proved the accuracy of a model (MIXTURE) providing a visual representation of both the quantity and the spatial arrangement of each morphological observation in comparison to the original Whole Slide Image (WSI) [20].

AI has been examined for the implementation of well-known limitations of diagnostic imaging, like chest X-rays. Evaluating the diagnostic accuracy in the screening of pulmonary tuberculosis, different results with different software were collected, giving high sensitivity for the AI detection of the disease [33]. Fanni et al. pointed to the solidity of AI-based software for the detection of lung nodules, automated flagging of positive cases of tuberculosis, and post-processing. They developed a digital bone suppression software that is able to produce highly accurate bone-suppressed images [34]. Numerous researchers have analyzed the impact of deep-learning image reconstruction, underlining the influence of AI in cancer screening, reducing image noise, and increasing the nodule detection rate and accuracy of chest CT images on ultra-low doses [35,36,37].

The significance of AI assistance has been highlighted as well in intensive care units (ICUs). Data showed that when physicians, even if beginners, were supported by the AI tool, their performance improved, exceeding the level of advanced users. Of course, the support of AI does not yield a significant enhancement for an expert ultrasonographer [38]. Automatic detection of abnormalities and pathological findings in LUS has been proposed [39], with good concordance between expert observers and automatic detection in a point-of-care setting [40,41]. This method has been proposed to make the evaluation of pulmonary edema easier. However, in a real-life study, physician evaluation was more specific despite both being highly sensitive [42]. Moreover, by combining murmur sound and LUS, a deep-learning algorithm was able to differentiate ILDs from COPD patients [43]. The use of AI in chest imaging was boosted by the COVID-19 pandemic [44], but automatic imaging evaluation was more on CT scans. Few experiences were performed on LUS with promising results [45]. Thus, we performed one of the few real-world evaluations of ILD patients using an automatic algorithm.

Our data indicate a concordance between the evaluation performed by an expert ultrasonographer and a beginner one who used automatic imaging processing. The close correlation we found between the Lung Staging measured with SensUS and the LUS score was relevant for a wide clinical use of automatic algorithms. In fact, the SensUS Lung device rises accordingly with the LUS score, indicating a good interchange. Furthermore, the correlations between SensUS values (e.g., Lung Staging) and lung function parameters shed light on the functional consequences of ILDs. Therefore, the SensUS Lung device may be useful for an ultrasound beginner to evaluate interstitial lung involvement rapidly and with good accuracy. Our results highlight the potential of the SensUS Lung device as a valuable tool for identifying ILDs in patients with compromised gas exchange in the lung.

In summary, the SensUS Lung device demonstrated its non-inferiority to LUS in ILD risk assessment and provided additional insights into the relationship between ILD severity, lung function, and Lung Staging. This information can help clinicians make better-informed decisions about the diagnosis and management of ILDs in their patients.

### 4.1. Limitations

Some limitations should be considered when interpreting results. First, the sample size is restricted. A larger and more diverse patient population would provide a more robust assessment of the automatic algorithm’s effectiveness. Moreover, the study was conducted in a single center, which might limit the generalizability of the findings to other healthcare settings and patient populations. Patients underwent HRCT as recommended by guidelines but only within three months before enrollment in this study. The LUS or SensUS Lung evaluation was not performed at the same time to minimize patient discomfort, so we chose not to compare these results to HRCT. Furthermore, the study focused on typical HRCT patterns (honeycombing, ground glass, fibrosis) for patient inclusion. Although these are common indicators of ILDs, the study did not explore the potential of automated algorithms in identifying atypical or rare ILD patterns for which the SensUS Lung device could be implemented. Thus, the SensUS Lung device, in its current state, should be regarded as a supportive tool for healthcare providers rather than a complete replacement for expert evaluation through LUS and HRCT. However, this is an exploratory evaluation in a real-life setting planned in a larger study as a starting point for a more complete algorithm upgrade.

### 4.2. Conclusions

Presently, AI or automated image processing can support but not replace LUS and HRCT performed by expert staff in monitoring patients. Our data suggest that AI algorithms may help non-expert physicians in LUS with results that are non-inferior to expert LUS despite it tending to overestimate ILD lesions. Therefore, algorithms like AI or automated image processing may support physicians (in particular, non-expert LUS sonographers) in daily clinical practice to monitor patients with ILDs. This device is simple to use, and it makes a fully automatic real-time analysis. However, it needs definite training in basic skills. In the future, AI and other automatic algorithms may be used to find patterns in enormous volumes of medical data, aiding in disease prediction and prevention before symptoms appear. More studies are needed to expand the clinical evidence base for the performance of these products.

## Figures and Tables

**Table 1:** Baseline features of 33 patients with ILDs considered to be all population and stratified according to the reduction or normal diffusion of lung Carbon Monoxide (DLCO) test. Comparison between these two groups was performed, and *p*-values are shown in the latter column.

Parameter	All Patients	Preserved DLCO	Reduced DLCO	*p*-Values
Demography				
Numbers of patients	33	17	16	
Age (years)	69 ± 17	62 ± 7	72 ± 16	Ns
Females/males	16 F/17 M	9 F/8 M	7 F/9 M	Ns
Weight (kg)	68.1 ± 13.66	67.6 ± 5.07	66.41 ± 13.54	Ns
Height (m)	1.64 ± 0.09	1.68 ± 0.07	1.63 ± 0.09	Ns
Body Mass Index (kg/m^2^)	25.0 ± 3.47	25.4 ± 3.39	24.6 ± 3.62	Ns
Smokers (former)	1 (9)	0 (3)	1 (6)	Ns
Clinical				
Hypertension	15	9	6	Ns
Diabetes (type 2)	7	4	3	Ns
Dyslipidemia	7	3	4	Ns
SARS-CoV-2 infection	14	9	5	Ns

Ns: not significant.

**Table 2:** Flow-volume spirometry value standardized for age, sex and BMI considering patients stratified according to the reduction or normal diffusion of lung Carbon Monoxide (DLCO) test. Comparison between these two groups was performed, and *p*-values are shown in the latter column.

Parameter	All Patients	Preserved DLCO	Reduced DLCO	*p*-Values
FEV1 (%)	101.60 ± 26.83	121.00 ± 9.66	104.18 ± 26.40	Ns
FVC (%)	101.50 ± 26.80	123.00 ± 10.22	99.90 ± 28.47	0.03
FEV1/FVC (%)	103.90 ± 13.61	100.00 ± 12.02	107.10 ± 12.02	Ns
FEF25 (%)	90.79 ± 42.29	118.80 ± 49.47	76.95 ± 35.95	Ns
FEF50 (%)	87.56 ± 30.53	97.00 ± 24.96	87.07 ± 30.20	Ns
FEF25–75 (%)	78.75 ± 35.74	79.66 ± 15.50	84.05 ± 45.05	Ns
FEF75 (%)	88.32 ± 39.53	97.80 ± 31.97	100.16 ± 41.40	Ns

Ns: not significant.

## Data Availability

The data are not publicly available due to ethical restrictions.

## References

[1] Sippel S., Muruganandan K., Levine A., Shah S. (2011). Review Article: Use of Ultrasound in the Developing World. Int. J. Emerg. Med..

[2] Vetrugno L., Mojoli F., Boero E., Berchialla P., Bignami E.G., Orso D., Cortegiani A., Forfori F., Corradi F., Cammarota G. (2022). Level of Diffusion and Training of Lung Ultrasound during the COVID-19 Pandemic—A National Online Italian Survey (ITALUS) from the Lung Ultrasound Working Group of the Italian Society of Anesthesia, Analgesia, Resuscitation, and Intensive Care (SIAARTI). Ultraschall Med..

[3] Dell’Aquila P., Raimondo P., Racanelli V., De Luca P., De Matteis S., Pistone A., Melodia R., Crudele L., Lomazzo D., Solimando A.G. (2022). Integrated Lung Ultrasound Score for Early Clinical Decision-Making in Patients with COVID-19: Results and Implications. Ultrasound J..

[4] Cicco S., Vacca A., Cariddi C., Carella R., Altamura G., Solimando A., Lauletta G., Pappagallo F., Cirulli A., Stragapede A. (2021). Imaging Evaluation of Pulmonary and Non-Ischaemic Cardiovascular Manifestations of COVID-19. Diagnostics.

[5] Cicco S., Marozzi M.S., Palumbo C.A., Sturdà E., Fusillo A., Scarilli F., Albanese F., Morelli C., Bavaro D.F., Diella L. (2023). Lung Ultrasound Is Useful for Evaluating Lung Damage in COVID-19 Patients Treated with Bamlanivimab and Etesevimab: A Single-Center Pilot Study. Medicina.

[6] Schäfer V.S., Recker F., Kretschmer E., Putensen C., Ehrentraut S.F., Staerk C., Fleckenstein T., Mayr A., Seibel A., Schewe J.-C. (2023). Lung Ultrasound in Predicting Outcomes in Patients with COVID-19 Treated with Extracorporeal Membrane Oxygenation. Viruses.

[7] Calamai I., Greco M., Bertolini G., Spina R. (2017). Current Adoption of Lung Ultrasound in Intensive Care Units: An Italian Multi-Center Survey. Minerva Anestesiol..

[8] Wallis A., Spinks K. (2015). The Diagnosis and Management of Interstitial Lung Diseases. BMJ.

[9] Luppi F., Sebastiani M., Salvarani C., Bendstrup E., Manfredi A. (2022). Acute Exacerbation of Interstitial Lung Disease Associated with Rheumatic Disease. Nat. Rev. Rheumatol..

[10] Ferro F., Delle Sedie A. (2018). The Use of Ultrasound for Assessing Interstitial Lung Involvement in Connective Tissue Diseases. Clin. Exp. Rheumatol..

[11] Wang Y., Gargani L., Barskova T., Furst D.E., Cerinic M.M. (2017). Usefulness of Lung Ultrasound B-Lines in Connective Tissue Disease-Associated Interstitial Lung Disease: A Literature Review. Arthritis Res. Ther..

[12] Demi L., Wolfram F., Klersy C., De Silvestri A., Ferretti V.V., Muller M., Miller D., Feletti F., Wełnicki M., Buda N. (2023). New International Guidelines and Consensus on the Use of Lung Ultrasound. J. Ultrasound Med..

[13] Umapathy V.R., Raj R.D.S., Yadav S., Munavarah S.A., Anandapandian P.A., Mary A.V., Padmavathy K., Akshay R., Rajkumar D.S.R., Anandapandian I.V. (2023). Perspective of Artificial Intelligence in Disease Diagnosis: A Review of Current and Future Endeavours in the Medical Field. Cureus.

[14] Miller D.D., Brown E.W. (2018). Artificial Intelligence in Medical Practice: The Question to the Answer?. Am. J. Med..

[15] Lombardi F.A., Franchini R., Morello R., Casciaro E., Ianniello S., Serra M., Satriano F., Mojoli F., Mongodi S., Pignatelli D. (2021). A New Standard Scoring for Interstitial Pneumonia Based on Quantitative Analysis of Ultrasonographic Data: A Study on COVID-19 Patients. Respir. Med..

[16] Marozzi M.S., Mancini F., Loponte L., Solimando A.G., Vacca A., Cicco S., Scholkmann F., LaManna J., Wolf U. (2023). Block of the Angiotensin Pathways Affects Flow-Volume Spirometry in Patients with SARS-CoV-2 Infection. Oxygen Transport to Tissue XLIV.

[17] Walsh S.L.F., Mackintosh J.A., Calandriello L., Silva M., Sverzellati N., Larici A.R., Humphries S.M., Lynch D.A., Jo H.E., Glaspole I. (2022). Deep Learning–Based Outcome Prediction in Progressive Fibrotic Lung Disease Using High-Resolution Computed Tomography. Am. J. Respir. Crit. Care Med..

[18] Cazzato G., Colagrande A., Cimmino A., Arezzo F., Loizzi V., Caporusso C., Marangio M., Foti C., Romita P., Lospalluti L. (2021). Artificial Intelligence in Dermatopathology: New Insights and Perspectives. Dermatopathology.

[19] Cazzato G., Massaro A., Colagrande A., Lettini T., Cicco S., Parente P., Nacchiero E., Lospalluti L., Cascardi E., Giudice G. (2022). Dermatopathology of Malignant Melanoma in the Era of Artificial Intelligence: A Single Institutional Experience. Diagnostics.

[20] Uegami W., Bychkov A., Ozasa M., Uehara K., Kataoka K., Johkoh T., Kondoh Y., Sakanashi H., Fukuoka J. (2022). MIXTURE of Human Expertise and Deep Learning—Developing an Explainable Model for Predicting Pathological Diagnosis and Survival in Patients with Interstitial Lung Disease. Mod. Pathol..

[21] Ienghong K., Cheung L.W., Tiamkao S., Bhudhisawasdi V., Apiratwarakul K. (2021). Development and Remodeling of Point-of-Care Ultrasound Education for Emergency Medicine Residents in Resource Limited Countries during the COVID-19 Pandemic. Tomography.

[22] Di Napoli A., Tagliente E., Pasquini L., Cipriano E., Pietrantonio F., Ortis P., Curti S., Boellis A., Stefanini T., Bernardini A. (2022). 3D CT-Inclusive Deep-Learning Model to Predict Mortality, ICU Admittance, and Intubation in COVID-19 Patients. J. Digit. Imaging.

[23] Fontanellaz M., Ebner L., Huber A., Peters A., Löbelenz L., Hourscht C., Klaus J., Munz J., Ruder T., Drakopoulos D. (2021). A Deep-Learning Diagnostic Support System for the Detection of COVID-19 Using Chest Radiographs: A Multireader Validation Study. Investig. Radiol..

[24] Anderson K.L., Fields J.M., Panebianco N.L., Jenq K.Y., Marin J., Dean A.J. (2013). Inter-Rater Reliability of Quantifying Pleural B-Lines Using Multiple Counting Methods. J. Ultrasound Med..

[25] Soldati G., Testa A., Sher S., Pignataro G., La Sala M., Silveri N.G. (2008). Occult Traumatic Pneumothorax. Chest.

[26] Stewart J.E., Goudie A., Mukherjee A., Dwivedi G. (2021). Artificial Intelligence-enhanced Echocardiography in the Emergency Department. Emerg. Med. Australas..

[27] Asch F.M., Poilvert N., Abraham T., Jankowski M., Cleve J., Adams M., Romano N., Hong H., Mor-Avi V., Martin R.P. (2019). Automated Echocardiographic Quantification of Left Ventricular Ejection Fraction Without Volume Measurements Using a Machine Learning Algorithm Mimicking a Human Expert. Circ. Cardiovasc. Imaging.

[28] Badano L.P., Boccalini F., Muraru D., Bianco L.D., Peluso D., Bellu R., Zoppellaro G., Iliceto S. (2012). Current Clinical Applications of Transthoracic Three-Dimensional Echocardiography. J. Cardiovasc. Ultrasound.

[29] Handa T. (2023). The Potential Role of Artificial Intelligence in the Clinical Practice of Interstitial Lung Disease. Respir. Investig..

[30] Dack E., Christe A., Fontanellaz M., Brigato L., Heverhagen J.T., Peters A.A., Huber A.T., Hoppe H., Mougiakakou S., Ebner L. (2023). Artificial Intelligence and Interstitial Lung Disease: Diagnosis and Prognosis. Investig. Radiol..

[31] Soffer S., Morgenthau A.S., Shimon O., Barash Y., Konen E., Glicksberg B.S., Klang E. (2022). Artificial Intelligence for Interstitial Lung Disease Analysis on Chest Computed Tomography: A Systematic Review. Acad. Radiol..

[32] Furukawa T., Oyama S., Yokota H., Kondoh Y., Kataoka K., Johkoh T., Fukuoka J., Hashimoto N., Sakamoto K., Shiratori Y. (2022). A Comprehensible Machine Learning Tool to Differentially Diagnose Idiopathic Pulmonary Fibrosis from Other Chronic Interstitial Lung Diseases. Respirology.

[33] Hua D., Nguyen K., Petrina N., Young N., Cho J.-G., Yap A., Poon S.K. (2023). Benchmarking the Diagnostic Test Accuracy of Certified AI Products for Screening Pulmonary Tuberculosis in Digital Chest Radiographs: Preliminary Evidence from a Rapid Review and Meta-Analysis. Int. J. Med. Inform..

[34] Fanni S.C., Marcucci A., Volpi F., Valentino S., Neri E., Romei C. (2023). Artificial Intelligence-Based Software with CE Mark for Chest X-Ray Interpretation: Opportunities and Challenges. Diagnostics.

[35] Jiang B., Li N., Shi X., Zhang S., Li J., De Bock G.H., Vliegenthart R., Xie X. (2022). Deep Learning Reconstruction Shows Better Lung Nodule Detection for Ultra–Low-Dose Chest CT. Radiology.

[36] Lee J.H., Lee D., Lu M.T., Raghu V.K., Park C.M., Goo J.M., Choi S.H., Kim H. (2022). Deep Learning to Optimize Candidate Selection for Lung Cancer CT Screening: Advancing the 2021 USPSTF Recommendations. Radiology.

[37] Ding Y., Zhang J., Zhuang W., Gao Z., Kuang K., Tian D., Deng C., Wu H., Chen R., Lu G. (2022). Improving the Efficiency of Identifying Malignant Pulmonary Nodules before Surgery via a Combination of Artificial Intelligence CT Image Recognition and Serum Autoantibodies. Eur. Radiol..

[38] Nhat P.T.H., Van Hao N., Tho P.V., Kerdegari H., Pisani L., Thu L.N.M., Phuong L.T., Duong H.T.H., Thuy D.B., McBride A. (2023). Clinical Benefit of AI-Assisted Lung Ultrasound in a Resource-Limited Intensive Care Unit. Crit. Care.

[39] Moshavegh R., Hansen K.L., Moller-Sorensen H., Nielsen M.B., Jensen J.A. (2019). Automatic Detection of B-Lines in In Vivo Lung Ultrasound. IEEE Trans. Ultrason. Ferroelect. Freq. Contr..

[40] Moore C.L., Wang J., Battisti A.J., Chen A., Fincke J., Wang A., Wagner M., Raju B., Baloescu C. (2022). Interobserver Agreement and Correlation of an Automated Algorithm for B-Line Identification and Quantification With Expert Sonologist Review in a Handheld Ultrasound Device. J. Ultrasound Med..

[41] Baloescu C., Rucki A.A., Chen A., Zahiri M., Ghoshal G., Wang J., Chew R., Kessler D., Chan D.K.I., Hicks B. (2023). Machine Learning Algorithm Detection of Confluent B-Lines. Ultrasound Med. Biol..

[42] Gottlieb M., Patel D., Viars M., Tsintolas J., Peksa G.D., Bailitz J. (2023). Comparison of Artificial Intelligence versus Real-Time Physician Assessment of Pulmonary Edema with Lung Ultrasound. Am. J. Emerg. Med..

[43] Siebert J.N., Hartley M.-A., Courvoisier D.S., Salamin M., Robotham L., Doenz J., Barazzone-Argiroffo C., Gervaix A., Bridevaux P.-O. (2023). Deep Learning Diagnostic and Severity-Stratification for Interstitial Lung Diseases and Chronic Obstructive Pulmonary Disease in Digital Lung Auscultations and Ultrasonography: Clinical Protocol for an Observational Case–Control Study. BMC Pulm. Med..

[44] Wang J., Yang X., Zhou B., Sohn J.J., Zhou J., Jacob J.T., Higgins K.A., Bradley J.D., Liu T. (2022). Review of Machine Learning in Lung Ultrasound in COVID-19 Pandemic. J. Imaging.

[45] Van Sloun R.J.G., Demi L. (2020). Localizing B-Lines in Lung Ultrasonography by Weakly Supervised Deep Learning, In-Vivo Results. IEEE J. Biomed. Health Inform..

